# Item pre-knowledge true prevalence in clinical anatomy - application of gated item response theory model

**DOI:** 10.1186/s12909-019-1710-z

**Published:** 2019-07-25

**Authors:** Milton Severo, Fernanda Silva-Pereira, Maria Amelia Ferreira, Magda Monteiro, Isabel Pereira

**Affiliations:** 1 Departamento de Ciências da Saúde Pública e Forenses e Educação Médica, Unidade de Educação Médica, Faculdade de Medicina da Universidade do Porto, Piso 6, Al. Prof. Hernâni Monteiro, 4200 – 319 Porto, Portugal; 2 Institute of Public Health at the University of Porto, Porto, Portugal; 3 Center for Research and Development in Mathematics and Applications (CIDMA), University of Aveiro, Aveiro, Portugal; 4 Águeda School of Technology and Management, Águeda, Portugal; 5 Department of Mathematics of University of Aveiro, Aveiro, Portugal

**Keywords:** Deterministic Gated item response theory model, Item pre-knowledge, Item sharing, Item exposure, Repeat items

## Abstract

**Background:**

Computer and paper examinations in our days are constructed from an item pool which is regularly updated. Given the way that exams are created, one of the major concerns is the security of the items that are being used in order to ensure a good estimation of abilities. The aim of this study is to measure the prevalence of item pre-knowledge in our medical school.

**Methods:**

The *Deterministic, Gated Item Response Theory Model* (DGM) was applied to estimate the prevalence of students who have had item pre-knowledge from six multiple choice examinations of the Clinical Anatomy course at the Faculty of Medicine of University of Porto. Each examination consisted of 100 items with an average of 200 students and 20% repeated items per examination. The estimation of the sensitivity and specificity was based on a simulation study. The sensitivity and specificity estimates, and apparent prevalence were used to estimate true prevalence of cheating students in the examinations under study.

**Results:**

The specificity in the DGM for different simulation scenarios was between 68 and 98%, while the sensitivity ranged from 60 to 91%. The apparent prevalence was between 0.0 and 3.4%, while the true prevalence ranged from 1.2 to 3.7%.

**Conclusions:**

The true prevalence was much lower compared to the students self-reported copying of responses from other students; however, it is important to keep monitoring the pre-knowledge prevalence in order to enforce measures in case an increase occurs.

**Electronic supplementary material:**

The online version of this article (10.1186/s12909-019-1710-z) contains supplementary material, which is available to authorized users.

## Background

Over a medical school course, it is very common to assess developed skills through multiple choice examinations [1]. An exam is constructed from an item pool which is regularly updated. Given the way that exams are created, one of the major concerns is the security of the items that are being used to ensure a good estimation of abilities. In some situations, students have item pre-knowledge either by over exposure or by item illicit access, and their item responses deviate from the underlying item response theory (IRT) by inflating their test scores [2].

Illicit access to items would be considered academic cheating. Academic cheating is defined as unethical or unauthorized academic activity, and is usually related to examinations [3]. A coordinated and purposeful exposure of items is very worrisome and would magnify examination scores for students who have gained examination pre-knowledge, while punishing honest students and consequently threaten the examination’s validity [4]. Additionally, item responses may move away from the subjacent IRT model [5].

Modeling potential behavior for students possessing prior item knowledge is further compounded by the issue of whether this knowledge is actually used to gain some advantages on the examination [4]. That is, modeling the impact of prior item knowledge is difficult because we need to identify disclosed items and we cannot disregard students who may have access to this information [6].

Several studies have shown that innocuous repeating of a small set of items within a larger examination had little impact on performance [2, 7, 8]. For example, in a national USA certification test in radiography, the same test or a different test form were assigned for the individuals that repeated the examination and indistinguishable score gains between the two groups were found [9]; a similar result was observed for the Medical Council of Canada Evaluating Examination [10]. Normally, the test-maker can control the proportion of reused items when assembling the test, however due to lack of time or economical pressure this is not always done.

The self-reported prevalence of item pre-knowledge was about 25%, while the self-reported prevalence of copying answers during an examination at least once during medical school has ranged from 52% [11] to 67% [12].

The most common way to detect copying answers or item pre-knowledge is using Classical Testing Theory (CTT) or Rasch IRT modeling to identify miss fitting response patterns. These miss fitting response patterns, especially on lower ability candidates on the examination overall, although not conclusive evidence of “cheating” per se, suggest that irregular behavior might have been engaged in order to achieve the correct responses on difficult questions (something we would not expect from low ability candidates). In this context, several classical statistics [3, 13–15] and software [16] have been developed to detect cheating on multiple-choice examinations.

Furthermore, several item pre-knowledge detection statistics have been recently developed [17, 18] and those that showed better efficiency were the posterior shift and the Shu Deterministic, Gated Item Response Theory Model (DGM) [19].

In 2013, Shu et al. proposed the DGM that classifies students as cheaters or non-cheaters according to score gain in the exposed items (e.g.: repeated items on previous examinations) compared to the non-exposed items (e.g.: new items) [18].

The proposed DGM consists of a two One-parameter Logistic (1-PL) model mixture [20–22] which classifies students into two groups, cheater and non-cheater by conditioning them to two types of items; the first type includes the items that are probably exposed, and the second type, the non-exposed items. The DGM allows item pre-knowledge detection through the analysis of the variation between students’ item pre-knowledge ability and their true ability.

Although, previous studies have measured the apparent prevalence (AP) (percentage of students classified as having item pre-knowledge), no studies have measured the true prevalence (TP) (percentage of students who truly have item pre-knowledge) as they did not take into account the sensitivity (SEN) and specificity (SPE) of the detection method.

In the case of high pre-knowledge item prevalence, the design of the examinations of Clinical Anatomy will need to be restructured.

The aim of this study was to estimate the item pre-knowledge true prevalence among medical students in the course of Clinical Anatomy at the Faculty of Medicine of University of Porto (FMUP) through the application of the DGM.

## Methods

All multiple choice examinations from the Clinical Anatomy course between 2008 and 2011 were analyzed to estimate the prevalence of students who had item pre-knowledge.

In each year, there were two final examinations which comprised a total of eight examinations. Each examination consisted of 100 standard multiple choice questions (MCQ) (five response options where only one was the correct answer), for a total of 800 items.

Each of the 100 items in each examination was compared with all other examination items in order to verify whether the item had been reused. The year 2008 was considered as the starting year and was excluded from the analysis because it did not contain any reused items. The items classified as reused were treated as exposed items, since students may have memorized items from a previously provided examination. The items used for the first time in the examination were treated as non-exposed items.

Initially, the data description was carried out using CTT in order to better comprehend the items’ characteristics; 1-PL and Two-parameter Logistic (2-PL) IRT models [20–22] were applied in order to validate the 1-PL model choice used in the DGM.

The 1-PL and 2-PL models were estimated using the marginal maximum likelihood estimation and the Expectation-Maximization (EM) algorithm [23, 24]. The chosen 1-PL model was the logistic model in which the discrimination parameter was estimated to be identical in all items.

In this study, the selected model was defined according to the Akaike Information Criterion (AIC) [25, 26], the Bayesian Information Criterion (BIC) [27, 28] and Convex Hull (CHull) method [29]. The model that better fits the data has the lowest AIC and BIC values, and the highest CHull value.

The difficulty (percentage of students who correctly answered the item) and discrimination index (biserial correlation between the item and the number of correct answers to the other items) of item examination were described using the mean and standard deviation (SD).

To assess whether there were significant differences between the examinations or number of repetitions, in the difficulty and discrimination indexes, mixed effect models were used with a fixed factor (examination or number of repetitions) and the item-level random intercept to account for the residual correlation within the same (reused) items.

Data were aggregated by item in order to eliminate the residual correlation within students that repeated the examinations; therefore, the previous model needed to include a student- level random intercept. The main reasons for aggregating data were data sparsity due to the reduced number of items reused and the small number of students that repeated the examinations; furthermore, item-level characteristics (e.g. the number of repetitions of the items) were the features of interest in this study.

The recommendations for the interpretation of the difficulty index suggest that values between 0 and 30% indicate a difficult item; values ranging from 31 to 80% imply an item with medium difficulty; values between 81 and 100% can be labeled as an easy question [30]. The recommendations for the interpretation of the discrimination index suggest five categories: values between − 1.00 and − 0.19 indicate negative discrimination; values ranging from − 0.20 to 0.19, weak discrimination; values between 0.20 e 0.29, sufficient discrimination; values from 0.30 to 0.39, good discrimination; and between 0.40 and 1.00, very good discrimination [31].

Cronbach’s alpha was used to assess the examination reliability. Recommendations suggest that examinations with 50 or more items have a good reliability if Cronbach’s alpha value is equal to or greater than 0.8 [32]. The alternative coefficient ω_h_ and ω_t_ of McDonald [33] was used as well to evaluate the reliability (general factor saturation and the inter-consistency, respectively) of the examinations.

### DGM

As referred previously, DGM is composed by a mixture of two 1-PL models which allows students to be classified into two groups. This classification takes into account the students results obtained in the secure and exposed items. Thus, DGM uses, on the one hand the true ability, *θ*_*tm*_, to characterize the real skill of the m^th^ student ,*m* = 1, …, *M*, and on the other hand, his/hers cheating ability, *θ*_*cm*_ to estimate cheating efficiency.

Therefore, DGM classifies each student with item pre-knowledge (cheater) or without item pre-knowledge (non-cheater) according to a specific threshold value.

Each item of the test is classified either as compromised or secure according to the fact that it is a reused item or not. Thus, for each item, *i,* the item exposure status, *G*_*i*_, is dichotomously defined as

$$ {G}_i=\left\{\begin{array}{c}1,\mathrm{compromised}\ \mathrm{item}\ i\ \\ {}0,\mathrm{secure}\ \mathrm{item}\ i\end{array}\right.\left(i=1,\dots, I\right) $$

Assuming that true and cheating abilities are known, student can be classified as a cheater if his/her true ability is lower than his/her cheating ability. Therefore, for each student is considered the dichotomous indicator variable *T*_*m*_

$$ {T}_m=\left\{\begin{array}{c}1,{\theta}_{tm}<{\theta}_{cm}\ \\ {}0,\mathrm{otherwise}\end{array}\right.\left(m=1,\dots, M\right) $$

where *T*_*m*_ = 1 represents that the *m* ^th^ examinee is a cheater.

The goal of conditioning the two item types is to use the information provided from the secured items to infer the level of item-compromise contained in the exposed items. The probability that the *m* ^th^ examinee answers correctly to the *i* ^th^ item is

$$ {P}_i\left({\theta}_{tm},{\theta}_{cm}\right)=P\left({y}_{mi}=1|{\theta}_{tm},{\theta}_{cm},{b}_i\right),\left(m=1,\dots, M,i=1,\dots, I\right), $$

where *b*_*i*_ represents the item difficulty index.

Both *G*_*i*_ and *T*_*m*_ are dichotomously defined, therefore, the DGM can be further broken down to four conditional models:

$$ P\left({y}_{mi}=1|{\theta}_{tm},{\theta}_{cm},{T}_m,{G}_i,{b}_i\right)=\left\{\begin{array}{c}\begin{array}{c}P\left({y}_{mi}=1|{\theta}_{tm},{b}_i\right)\ \mathrm{for}\ {T}_m=0,{G}_i=0\\ {}P\left({y}_{mi}=1|{\theta}_{tm},{b}_i\right)\ \mathrm{for}\ {T}_m=1,{G}_i=0\\ {}P\left({y}_{mi}=1|{\theta}_{cm},{b}_i\right)\ \mathrm{for}\ {T}_m=1,{G}_i=1\end{array}\\ {}P\left({y}_{mi}=1|{\theta}_{tm},{b}_i\right)\ \mathrm{for}\ {T}_m=0,{G}_i=1\end{array}\right. $$

When the student is classified as a non-cheater, *T*_*m*_ = 0, the responses to all items are based only on his/her true ability, *θ*_*tm*_, and therefore do not depend on *θ*_*cm*_. However, when *T*_*m*_ = 1, that is, for students that are cheaters, it is necessary to take into account whether the items are exposed or not. Student answers to the unexposed items (*G* = 0) are based on their true ability (*θ*_*tm*_), while responses to the exposed items (*G* = 1) are based on their cheating ability (*θ*_*cm*_). Accordingly, cheating ability only influences the response probability of cheating students in the exposed items.

Taking into consideration the *G*_*i*_ and *T*_*m*_ values, the probability of the m^th^ student correctly answering item *i* can be written as a unique expression

$$ P\left({y}_{mi}=1|{\theta}_{tm},{\theta}_{cm},{T}_m,{G}_i,{b}_i\right)=P{\left({y}_{mi}=1|{\theta}_{tm},{b}_i\right)}^{1-{T}_m}\times {\left[\left(1-{G}_i\ \right)P\left({y}_{mi}=1|{\theta}_{tm},{b}_i\right)+{G}_iP\left({y}_{mi}=1|{\theta}_{cm},{b}_i\right)\right]}^{T_m} $$

emphasizing the mixture structure of the model used.

In order to discriminate if the student is classified as cheater or non-cheater, it is necessary to fix a value representing the cut-off point. This threshold was defined according to the probability of a student being a cheater (T = 1), *P*_*c*_ (0 < *P*_*c*_ < 1), by the DGM. Shu et al [11] used the fixed value of 90% as the cut-off point *P*_*c*_, while in the present study, we also used a classification tree to identify the best cut-off point value of *P*_*c*_ to classify students with or without item pre-knowledge. Classification trees are a statistical method used to construct binary trees, by successive divisions of data according to a rule that divides the data into groups as uniform as possible [34]. Homogeneity between the two constituted subgroups is defined by impurity – a measure that takes the zero value in completely homogeneous subgroups. In classification trees (the response variable is qualitative); impurity can be measured by the amount of entropy, which must be minimized since it measures heterogeneity within groups. Thus, the criterion used to choose the best cut-off point from all possible cut-off point values was the one that minimized entropy.

### Simulation study

This subsection aims to describe the conditions of the simulation study that supported the analysis of sensitivity and specificity of the DGM as well as the best choice of the cut-off point that distinguishes cheaters from non-cheaters.

The simulation study was carried out considering the closest conditions to the ones verified in the Clinical Anatomy course examinations. In real data, there were an average of 20 reused items and 200 students per examination, and those values were used in the simulation study. The simulation study must take into account the item pre-knowledge characteristics, including the proportion of item pre-knowledge and the effectiveness of item pre-knowledge. The proportion of item pre-knowledge refers to the percentage of students who have pre-knowledge of the exposed items. The effectiveness of item pre-knowledge is the effective score gain as a result of prior knowledge of the exposed items. According to the score gain level, the most effective students (high-effective) obtain the most effective gain and low effective (low-effective) obtain a lower effective gain. We considered four scenarios with four levels of proportion of item pre-knowledge, 5, 10, 35 and 70%, and two levels of cheating efficacy of item pre-knowledge, high-effective and low-effective. For each of the scenarios, we simulated 100 replicates.

The items’ difficulty (*b*) was simulated according to a standard normal distribution. The student’s true ability (*θ*_*t*_) was simulated according to the standardized normal distribution, *N*(0, 1) and student’s cheating ability (*θ*_*c*_) was obtained by the sum of the effective score gain, (*∆*), to true ability. In a non-cheating student, the effective gain is zero, while for a cheating student; it is simulated from a beta distribution. When the cheating category is high-effective, the score gain is characterized by *Beta*(9, 4) ∗ 3 and when it is low-effective, it is simulated according to *Beta*(5, 5) ∗ 3.

Thus, we can summarize the distributions used in the simulation of the parameters related to items and students of the DGM as

$$ {\displaystyle \begin{array}{c}{\theta}_t,b\sim N\left(0,1\right),\\ {}{\theta}_c={\theta}_t+\Delta , \end{array}} $$

with *∆* = 0 for the non-cheater, *∆*~*Beta*(9, 4) ∗ 3 for the cheater high-effective and *∆*~*Beta*(5, 5) ∗ 3 for the cheater low-effective.

Let *Y*_*mi*_, *m* = 1, …, 200, *i* = 1, …, 100, be the response of student *m* to item *i*. *Y*_*mi*_ were generated using the equations

$$ P\left({Y}_{mi}=1\right)={P}_i\left({\theta}_{cm}\right)=\frac{1}{1+{e}^{\theta_{cm}-{b}_i}} $$

for the exposed items and cheaters, and

$$ P\left({Y}_{mi}=1\right)={P}_i\left({\theta}_{tm}\right)=\frac{1}{1+{e}^{\theta_{tm}-{b}_i}} $$

for all other cases.

### Estimation of the DGM

The parameters of the DGM were estimated using Markov chain Monte Carlo (MCMC) [35, 36] methods through Gibbs algorithm [37]. The following prior distributions were considered:

$$ {\displaystyle \begin{array}{c}{Y}_{mi}\sim Bernoulli\left({P}_i\left({\theta}_{cm}\right)\right),\\ {}{\theta}_{tm},b\sim N\left(0,1\right),\\ {}{\theta}_{cm}\sim N\left(1,2\right),\\ {}{T}_m=1\kern0.75em \mathrm{when}\kern0.5em {\theta}_{tm}<{\theta}_{cm.}\end{array}} $$

These variables are i.i.d for *m* = 1, …, 200, *i* = 1, …, 100

Since the distributions of *θ*_*tm*_ and *θ*_*cm*_ do not depend on the considered student, for simplification, considerer *θ*_*tm*_ = *θ*_*t*_ and *θ*_*cm*_ = *θ*_*c*_. WinBUGS’ DGM commands are available in Additional file 1.

For each DGM, sample parameters were generated, with dimension 110,000 from the posterior distribution, which include a burn-in period of 10,000 observations to ensure the convergence of Markov chains in the sampling process. Only observation parameters with a 100 iterations jump in order to obtain a sample, with dimension 1,000, of approximately uncorrelated observations were stored.

### Estimation of the true prevalence

In real data, we do not know if a student is a cheater or not. When we apply a DGM, it tells us which students were classified by the model as cheaters (positive test). The percentage of those students is referred to as the apparent prevalence (AP) and is obtained by

$$ AP\ \left(\%\right)=\frac{\# positives}{\# total\ students}\times 100 $$

We want to know the percentage of students who are truly cheaters; the true prevalence (TP) [38] is

$$ TP\ \left(\%\right)=\frac{\# cheaters}{\# total\ students}\times 100 $$

A Bayesian approach can be used to estimate the TP [39] using the following relationship with the AP and taking into account the sensitivity (SEN) and specificity (SPE) of the DGM.

$$ AP= TP\times SEN+\left(1- TP\right)\times \left(1- SPE\right). $$

The SEN is the percentage of students who were correctly classified as cheaters and the SPE is the percentage of students who were correctly classified as non-cheaters [40].

To obtain the TP, we used the SEN and the SPE means and SD computed in the simulation study. The minimum SEN and SPE for the uniform distribution were fixed for the DGM classification as the minimum and the maximum *mean* for all scenarios in the simulation study.

The R software [41] was used for statistical analysis and for programming.

Furthermore, the estimation of parameters was performed by Gibbs algorithm, implemented in WinBUGS through the R2WinBUGS package [42], the rpart package [43] for the classification trees, the ltm package [44] to see which model best fit the data, and for the algorithm distributions display and convergence study, we used the coda packages [45] and mcmcplots [46].

## Results

### Simulation study

The SEN and SPE for the cut-off point of 90% were obtained by computing the 100 replicates of the simulations for the different scenarios showed in Table 1. The SPE was higher than 90%, while the SEN ranged from 60.3 to 90.7%.

**Table 1:** Specificity, sensitivity, positive and negative predictive value in each scenario of the simulation study using the cut-off value of 90%

Proportion (True prevalence)	5%	5%	10%	10%	35%	35%	70%	70%
Cheating efficacy	High	Low	High	Low	High	Low	High	Low
	Mean (SD)	Mean (SD)	Mean (SD)	Mean (SD)	Mean (SD)	Mean (SD)	Mean (SD)	Mean (SD)
Specificity (%)	77.82 (2.74)	78.16 (3.76)	81.65 (3.28)	81.46 (3.41)	94.41 (3.02)	92.33 (2.83)	98.00 (1.80)	97.65 (2.14)
Sensitivity (%)	69.78 (14.52)	60.30 (15.73)	81.35 (7.88)	69.35 (12.16)	83.75 (15.78)	83.35 (7.28)	90.75 (2.48)	68.99 (11.40)
Positive predictive value (%)	14.84 (6.37)	12.71 (3.48)	34.07 (7.92)	29.54 (5.57)	88.42 (10.31)	85.30 (4.50)	99.07 (0.83)	98.66 (1.20)
Negative predictive value (%)	96.90 (8.59)	96.66 (7.02)	97.18 (2.83)	96.00 (1.55)	92.02 (6.77)	89.88 (3.13)	82.30 (4.13)	58.31 (7.65)
Model absolute agreement (%)	77.44 (2.81)	76.97 (3.37)	81.16 (5.56)	80.16 (3.36)	90.54 (4.17)	88.21 (2.71)	92.93 (1.75)	77.59 (7.71)
Apparent Prevalence	24.52 (2.7)	24.07 (3.2)	24.7 (2.9)	23.7 (3.1)	32.8 (7.2)	33.2 (3.5)	64.1 (1.9)	49.0 (8.3)
Cohen’s Kappa (%)	17.22 (6.86)	15.07 (9.56)	38.32 (6.96)	31.64 (8.46)	78.43 (10.68)	73.76 (6.29)	84.21 (3.71)	56.26 (11.41)
Best Cut-off point^a^	99.9%	100%	98.6%	98.8%	86.8%	91.4%	79.3%	65.9%

^a^Best Cut-off point estimate by a classification tree

The AP in all scenarios was different compared to the TP (Table 1).

The simulation study showed that for high prevalence, the cut-off value should be decreased, and for low prevalence, the cut-off value should be increased.

**Fig. 1:**
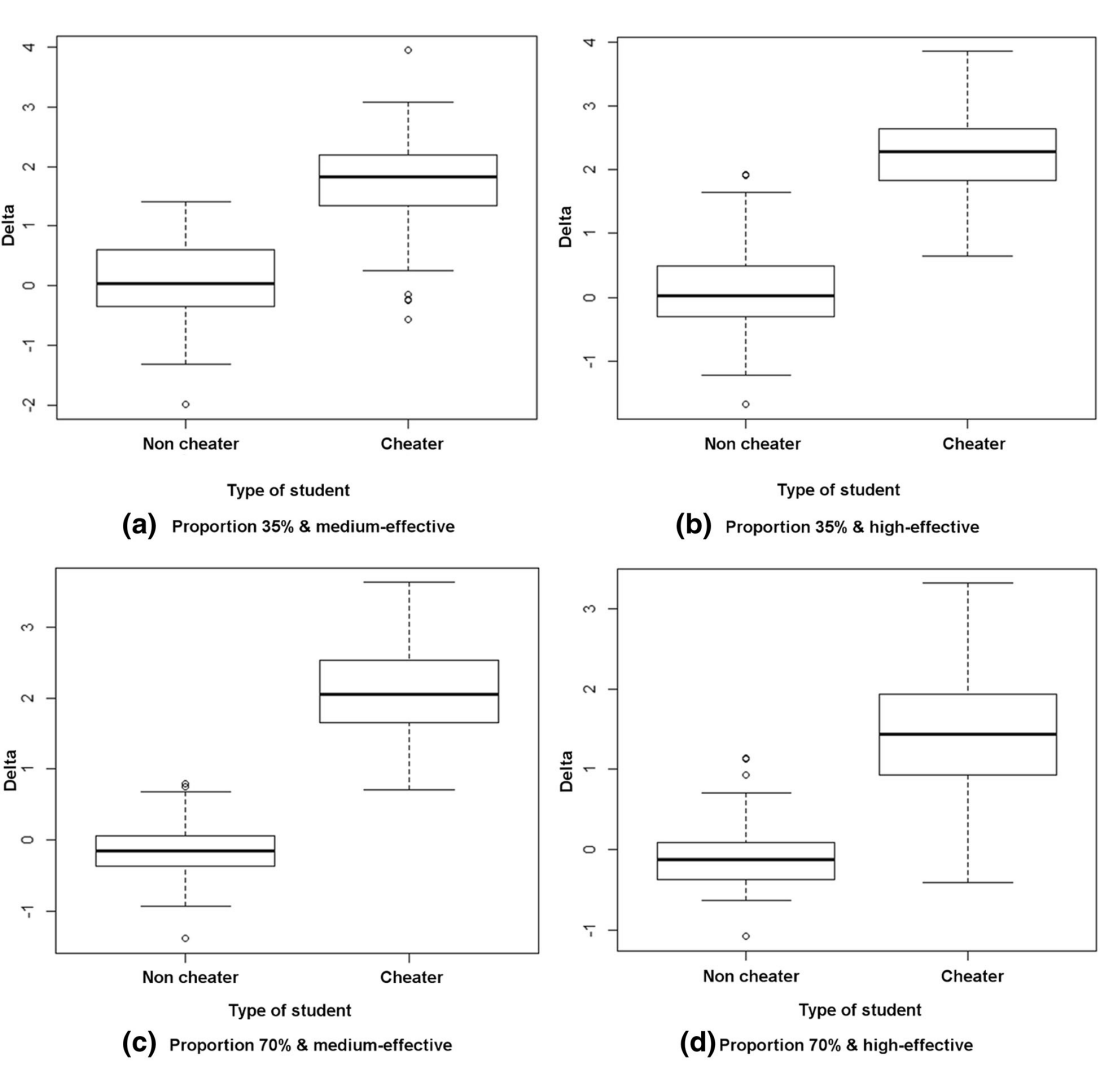
Example of the effective gain of the cheating versus non-cheating students. **a** Proportion 35% & medium-effective. **b** Proportion 35% & high-effective. **c** Proportion 70% & medium-effective. **d** Proportion 70% & high-effective

Figure 1 presents the estimated gain for each one of the scenarios. We can observe that a cheating student obtains a much higher effective score gain than a non-cheating student. For the non-cheating student, the score gain is very close to 0. If we analyze Fig. 1a and b we can observe that for the same proportion of item pre-knowledge (35%), students obtain a higher effective score gain when it is high-effective; the same happens for the proportion of item pre-knowledge (70%) (Fig. 1c and d).

### Application to real data

#### Data description

A total of 1008 students completed the examination between 2008 and 2011, from those 774 (76.8%), 218 (21.6%), 14 (1.4%) and 2 (0.2%) completed the examination 1, 2, 3 and 4 times, respectively.

Table 2 shows the number of reused items, the number of items reuses, the students’ mean score, and the items’ difficulty and discrimination mean levels and respective Cronbach’s alpha and McDonald’s ω_h_ and ω_t_ for each examination.

**Table 2:** Data description according to CTT

Year	Period	N	Reused (N)	Difficulty^a,c^ Mean (SD)	Discrimination^b,c^ Mean (SD)	Cronbach’s Alpha	ω_h_	ω_t_
2008	1	217	0	0.57 (0.18)	0.37 (0.19)	0.88	0.46	0.89
	2	123	4	0.63 (0.20)	0.32 (0.17)	0.86	0.51	0.87
2009	1	208	13	0.66 (0.20)	0.34 (0.13)	0.88	0.48	0.88
	2	113	6	0.64 (0.21)	0.37 (0.16)	0.89	0.38	0.90
2010	1	192	26	0.64 (0.20)	0.30 (0.14)	0.86	0.44	0.87
	2	116	13	0.59 (0.21)	0.32 (0.15)	0.87	0.41	0.88
2011	1	243	21	0.62 (0.22)	0.37 (0.17)	0.89	0.56	0.90
	2	48	14	0.59 (0.20)	0.37 (0.19)	0.90	0.36	0.91

^a^% of students who correctly answered the item

^b^ Biserial correlation between the item and the number of correct answers to the other items

^c^ To assess whether there were significant differences between the examinations, in the difficulty and discrimination indexes, mixed effect models were used with a fixed factor (examination) and the item-level random intercept to account for the residual correlation within the same (reused) items

From a total of 800 items, 84 (10.5%) were reused once, 13 (1.62%) twice, and the percentage of repetitions ranged from 4 to 26%. The mean items’ difficulty index was between 0.57 and 0.66, there were statistically significant differences in the difficulty index by examination (*p* = 0.0471), and all examinations showed a medium difficulty level. The mean items’ discrimination index ranged from 0.30 and 0.37, and there were statistically significant differences in discrimination index by examination (*p* = 0.008); however, all examinations presented good discrimination. Cronbach’s alpha was above 0.8 in all examinations, which showed that all examinations have a good reliability. The ω_t_ showed high internal consistency and the ω_h_ moderated the general factor saturation for all examinations.

The index of difficulty increased 3.5% (*p* = 0.013) in the first repetition and 6.9% (*p* = 0.036) in the second repetition compared to the first time, meaning that with repetitions, the items were easier for the students (Table 3).

**Table 3:** Mixed effect models to measure repetition effects on difficulty and discrimination indices using CTT

	Difficulty index			Discrimination index		
	$\hat{\beta}$	95%CI	*p*	$\hat{\beta}$	95%CI	*p*
	Model 1^a^			Model 2^a^		
Intercept	0.613	[0.598,0.629]	< 0.001	0.337	[0.325,0.348]	< 0.001
1st repetition	0.035	[0.008,0.063]	0.013	0.026	[−0.008,0.060]	0.135
2nd repetition	0.069	[0.005,0.133]	0.036	0.009	[−0.073,0.092]	0.820

^a^To assess whether there were significant differences between the number of repetitions, in the difficulty and discrimination indexes, mixed effect models were used with a fixed factor for repetitions and the item-level random intercept to account for the residual correlation within the same (reused) items

#### Goodness-of-fit of 1-PL model

In order to assess if we could use the 1-PL model to fit the data we compared the 1-PL and 2-PL models to verify which one gives the best fit to the real data. Table 4 presents a summary of the goodness-of-fit index for year and period. Using BIC and CHull, the 1-PL model better fits the data in the eight examinations. Using AIC, in five of the eight examinations, the model that fits better is the 2-PL model (Table 4).

**Table 4:** The goodness-of-fit of the 1-PL and 2-PL models to real data by year and period

Year	Period	Model	Log-Lik	AIC^a^	BIC^a^	CHull^a^
2008	1	1-PL	− 12685.50	25573.09	**25914.46**	**23.58**
		2-PL	− 12536.60	**25473.24**	26149.22	1.09
	2	1-PL	− 6707.50	13617.01	**13901.04**	**17.02**
		2-PL	− 6592.09	**13584.17**	14146.61	1.13
2009	1	1-PL	− 10729.40	21660.88	**21997.97**	**22.49**
		2-PL	− 10619.00	**21637.89**	22305.40	0.97
	2	1-PL	− 5862.56	**11927.13**	**12202.59**	**8.81**
		2-PL	− 5774.92	11949.85	12495.32	0.81
2010	1	1-PL	− 10321.70	20845.34	**21174.34**	**29.82**
		2-PL	− 10207.60	**20815.13**	21466.62	1.11
	2	1-PL	− 6499.01	**13200.01**	**13478.12**	**18.40**
		2-PL	− 6403.77	13207.53	13758.25	1.05
2011	1	1-PL	− 12838.10	25878.19	**26230.99**	**10.10**
		2-PL	− 12629.10	**25658.23**	26356.84	2.27
	2	1-PL	− 2687.06	**5576.12**	**5765.11**	**5.34**
		2-PL	− 2614.77	5629.54	6003.78	0.84

^a^Bold values identifies the best model according to each criterion

#### Item pre-knowledge prevalence

The DGM estimated that the AP ranged from 0.00 to 3.30%, and the TP after using the information SEN and SPE from the simulation study was between 1.20 and 3.70% for all examinations (Table 5).

**Table 5:** Mean and SD values obtained for the apparent prevalence and the true prevalence for the examinations between 2009 and 2011

Year	Period	AP^a^	TP^b^
		Mean (SD)	Mean (SD)
2009	1	2.2 (0.2)	1.5 (1.3)
2009	2	0.0 (0.0)	1.2 (1.2)
2010	1	2.5 (0.2)	1.6 (1.4)
2010	2	3.3 (0.5)	2.6 (2.2)
2011	1	3.4 (0.2)	1.8 (1.5)
2011	2	2.1 (0.0)	3.7 (3.4)

^a^*AP* % of students considered cheater (above cut-off point)

^b^*TP* True prevalence

**Fig. 2:**
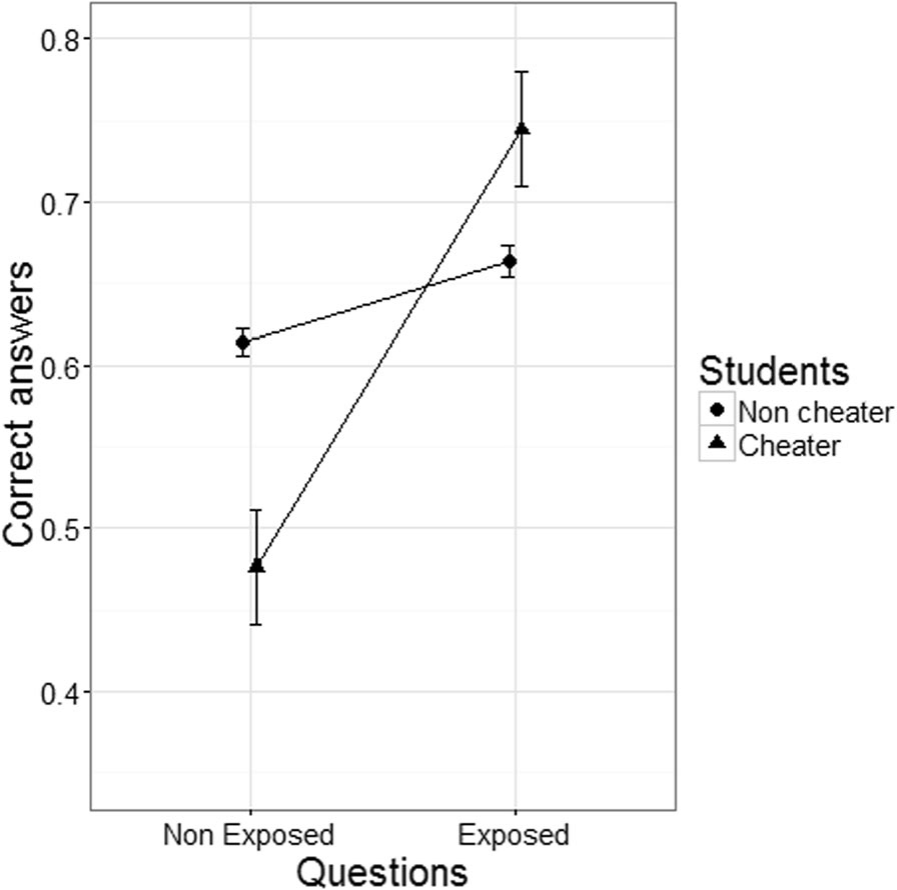
Percentage of correct answers by item and student type 2011 examination period 1

This situation happens in all studied examinations and can be seen in Fig. 2, where for students considered not cheaters regardless of item exposure or not, the percentage of the students correct answers practically does not change; the same cannot be said for students considered cheaters. In this case, the percentage of correct answers in the exposed items increases very significantly when compared to the percentage of correct answers in the unexposed items. This was expected considering that the DGM model more easily detects the students with item pre-knowledge with low ability. Those students will have a high gain in the number of correct answers compared to students with high ability where the gain would be smaller, and consequently more difficult to detect. Additionally, these students (with low ability) will be more effective compared to the high ability students in the exposed items, since the main focus will usually be items memorization from past examinations compared to high ability students that use all types of information and so will not be so effective in memorizing items.

Focusing only on the non-exposed items, there is a considerable difference between the two groups of students revealing differences in their true skills that can also be explained by the arguments referred to above.

## Discussion

In this work, the DGM was applied to six multiple choice examinations of FMUP’s Clinical Anatomy course. The proportion of pre-knowledge items in the analyzed examinations ranged from 1.2 to 3.7%, that is, in this course, the proportion of item pre-knowledge is low compared to the self-reported prevalence of copying answers during an examination at least once during medical school, which has ranged from 52% [8] to 67% [9] and the self-reported prevalence of item pre-knowledge was about 25%. When compared to the prevalence using detection statistics for copying answers, the prevalence was high, for example, in 11 examinations held by the Royal College of Pediatrics and Child Health, there was a prevalence of 0.1% [3]. In a low-stake test for measuring student proficiency in Grade 4 English, the prevalence of item pre-knowledge was about 9% [11].

The low prevalence in this study may firstly be due to the fact that students do little study by previously provided examinations or to the fact that students study by the previously provided examinations but also simultaneously through other sources and therefore there is no big difference between the students’ true ability and their cheating ability because these students have a high true ability. The second hypothesis is supported by the fact that no differences in the difficulty index were detected between exposed and unexposed items within the examination itself, however, over the years, significant differences in the difficulty index were detected and exposed items became increasingly easy.

This is the first study that tries to measure the TP of item pre-knowledge; other studies have used the AP determined through a diagnostic test, which will differ from the TP. In our case, we showed that the “apparent” prevalence would underestimate/overestimate the TP depending on the examination.

The simulation study was required to assess the effectiveness of the DGM under the same conditions of the real data and the DGM was applied to the real data in order to estimate the TP of cheating students per examination. The simulation study showed the effectiveness of the DGM when the number of items per test is high (100), the proportion of the exposed items is low (20%) and the number of students is small (200). The absolute agreement of the DGM with these conditions was more than 76%. In the previous study by Shu, the effectiveness of the DGM was studied for an examination with 40 items, a proportion of committed items higher or equal to 30% and 15,000 students, and the cut-off point was set at 0.9. Our study showed that in the case of a high pre-knowledge prevalence, the SEN was lower compared to the SPE, thus increasing the bias between the AP and the TP. Changing the cut-off value from 90% to lower values would decrease the difference between the SEN and the SPE, thus decreasing the bias between the AP and the TP (data not shown). If the test-maker has a priori information that the pre-knowledge prevalence is high, they should lower the threshold in order to use the AP as an estimate of the TP.

One possible constraint of this study was the fact that the 1-PL model used by the DGM could not fit the real data and diminished the diagnostic capacity; however, the BIC showed that the 1-PL model had a better fit compared to the 2-PL model.

The analyzed examinations had a medium difficulty, good discrimination and good reliability scores using both the CTT and the IRT, showing that the low prevalence of item pre-knowledge did not have a large impact on the quality of the examinations.

Moreover, it is worthwhile to mention that one restriction of the present work is the small scale of the study. Surely, it would be of interest to apply DGM in a larger scale with the increase of response sample size and the inclusion of clinical courses in which item re-usage is more common. This remains a topic for future research.

## Conclusions

The DGM has proved effective in item pre-knowledge detection and the prevalence of item pre-knowledge is low. The simulation study showed that the DGM underestimates/overestimates the TP.

Thus, the threshold established should be lower in the case of high pre-knowledge prevalence in order to diminish the bias between the AP and TP.

We will keep monitoring the pre-knowledge prevalence in order to take measures in the case of an increase. These could be by the exclusion of exposed items for the next examinations or to provide seminars to increase the awareness of this problem.

## Additional file

Additional file 1: WinBUGS DGM Model Commands. (DOCX 17 kb)

## Data Availability

The datasets used and/or analysed during the current study are available from the corresponding author on reasonable request.

## Abbreviations

1-PL: One-parameter Logistic
2-PL: Two-parameter Logistic
AIC: Akaike Information Criterion
AP: Apparent Prevalence
BIC: Bayesian Information Criterion
CHull: Convex Hull
CI: Confidence Interval
CTT: Classical Test Theory
DGM: Deterministic, Gated Item Response Theory Model
EM: Expectation-Maximization
FMUP: Faculty of Medicine of the University of Porto
IRT: Item response theory
Log-Lik: Log-likelihood
MCMC: Markov chain Monte Carlo
MCQ: Multiple choice questions
SD: Standard deviation
SEN: Sensitivity
SPE: Specificity
TP: True Prevalence
