# Supplementary material for: Item pre-knowledge true prevalence in clinical anatomy - application of gated item response theory model
Source: BMC Med Educ. 2019 Jul 25;19:284. doi: 10.1186/s12909-019-1710-z (PMC6659220; doi:10.1186/s12909-019-1710-z)
Supplement: Supplementary file 1 — WinBUGS DGM Model Commands. (DOCX 17 kb) [file 12909_2019_1710_MOESM1_ESM.docx]

**Additional file 1**

**WinBUGS DGM Model Commands**

model

{

# Calculate individual (binary) responses to each test from

multinomial data

for (j in 1 : N) {

for (k in 1 : T) {

r[j, k] <- x[j, k]

}

}

# Rasch model

for (j in 1 : N) {

for (k in 1 : T) {

logit(p[j, k]) <- (step(k-T1-1)*

step(thetac[j]-theta[j]))*

(thetac[j] - alpha[k])

+(1-step(k-T1-1)*step(thetac[j]-theta[j]))*

(theta[j] - alpha[k])

r[j, k] ~ dbern(p[j, k])

}

thetac[j] ~ dnorm(1, 0.5)

theta[j] ~ dnorm(0, 1)

}

# Priors

for (k in 1:T) {

alpha[k] ~ dnorm(0, 1);

}
